# The President’s Emergency Plan for AIDS Relief and adult mortality: A replication study of HIV development assistance effects in Sub-Saharan African countries

**DOI:** 10.1371/journal.pone.0233948

**Published:** 2020-10-26

**Authors:** Nicholas A. Hein, Danstan S. Bagenda, Jiangtao Luo

**Affiliations:** 1 Department of Biostatistics, College of Public Health, University of Nebraska Medical Center, Omaha, NE, United States of America; 2 Department of Anesthesiology College of Medicine, University of Nebraska Medical Center, Omaha, NE, United States of America; 3 EVMS-Sentara Healthcare Analytics and Delivery Science Institute & Department of Internal Medicine, Eastern Virginia Medical School, Norfolk, VA, United States of America; Kobenhavns Universitet, DENMARK

## Abstract

The US budget for global health funding, which was by far the largest of similar funding in the world, increased from US $1.3 billion in 2001 to more than US $10 billion in recent years. More than 54% of this funding was allocated to the Global Fund to Fight HIV/AIDS through the US President’s Emergency Plan for AIDS Relief (PEPFAR) in Africa. However, recent studies indicate contradictory results regarding the effectiveness of PEPFAR. One by Bendavid, Holmes, Bhattacharya, and Miller shows positive effects of PEPFAR in reducing adult mortality in Africa, while another by Duber, Coates, Szekeras, Kaji, and Lewis finds that there are no significant differences in reducing adult mortality in countries that received PEPFAR funding vs countries that did not. Due to their potential impact on policy decisions regarding critical global health funding, we wanted to assess why the results are discrepant. To do this, we replicated the Bendavid study. The replication provides verification that the study replicable and that the analytic choices of the authors are robust to different assumptions or restrictions. This allows us to assess the different choices and data available to the two research groups and draw some conclusions about why the results may be different. Then, focusing on two of the prominently discrepant studies, i.e., the Bendavid study (1998–2008) and the Duber study (2000–2006), we establish why the two studies are in disagreement. We apply appropriate individual-level and country-level analytical methodology as used by Bendavid over the analytical time period used for the Duber study (2000–2006), which originally focused on nationally aggregated data and differed in some key focus countries. For our first objective, we replicated the original Bendavid study findings and our findings support their conclusion that between 1998–2008 all-cause mortality decreased significantly more (OR = 0.84, CI, 0.72–0.99) in countries that implemented PEPFAR. For our second objective (Bendavid’s data and methodology applied to Duber’s study period), we found reduction in all cause adult mortality to be borderline insignificant (OR = 0.87 CI, 0.75–1.01, p = 0.06), most possibly reflecting the abbreviated fewer number of events and sample size over a shorter period. Therefore, our overall analyses are consistent with the conclusion of positive impact of the PEPFAR program in reducing adult mortality. We believe that the discrepancy observed in the original studies mainly a reflection of shortcomings in the analytical approach necessitated by the Duber study’s nationally aggregated dataset or “may reflect a lack of data quality” in the Duber study (Duber, et al. 2010).

## 1. Introduction

There was a significant increase in the US budget for global health funding, starting in 2001 [1]. Since policy decisions often hinge on whether aid allocation had a significant and intended impact, understanding the relationships between these factors is of critical importance [2]. A considerable amount of global health funding had been used for the Global Fund to Fight AIDS through the United States President’s Emergency Plan for AIDS Relief (PEPFAR) in Africa, but the estimates of its impact has been mixed [3–8]. PEPFAR began its first full year of funding in 2003 and provided funding to 15 focus countries for the delivery of antiretroviral therapy and HIV prevention programs (PEPFAR 2015). Funding allocated to PEPFAR countries increased dramatically between 2001 and 2010 [5], but the effectiveness of the increased funding to these focus countries on adult mortality was under-studied. Previous studies addressing this question either showed no effect of increased PEPFAR funding on adult mortality during a relatively circumscribed time frame, 2000 to 2006 [4], or used estimates with modeled data of mortality rates [9]. In this study, we focus on two studies [3] and [4] with the aim of understanding why they showed differing results regarding the impact of PEPFAR funding. We selected Bendavid, Holmes, Bhattacharya, and Miller (BHBM) for primary analysis because the quality of its data and longer timeframe and used the timeframe from [4] to assess why [3] was able to show significant results while [4] was not.

The BHBM study, “HIV Development Assistance and Adult Mortality in Africa,” [3] sought to determine the difference of adult mortality between PEPFAR focus and non-focus countries using a broader time frame as well as survey data from individuals to more directly measure mortality. BHBM performed two primary analyses: (1) a cross-country comparison of adult mortality between 1998 and 2008 in 9 African countries receiving PEPFAR funding (focus countries) and 18 African countries that did not receive funding (non-focus countries), and (2) a within-country comparison of the intensity of PEPFAR implementation and adult mortality in 22 districts of Tanzania and 30 districts of Rwanda. The main finding of the study is that adult mortality declined more dramatically in countries receiving PEPFAR funding compared to countries not receiving PEPFAR funding. Specifically, in 2003, prior to the increase in funding, the age-adjusted adult mortality was 8.3 per 1,000 (95% confidence interval [CI], 8.0–8.6) in PEPFAR countries and 8.5 per 1,000 adults (95% CI, 8.3–8.7) in non-PEPFAR countries. In 2008, the adult mortality in PEPFAR countries was 4.1 per 1,000 (95% CI, 3.6–4.6) compared with 6.9 per 1,000 adults (95% CI, 6.3–7.5) in countries not receiving PEPFAR funding. Unfortunately, BHBM is unable to distinguish between effects on all-cause adult mortality or effects solely on HIV-related mortality. However, BHBM [3] identifies two additional factors associated with lower adult mortality–the educational level of the female respondents to the individual household surveys and the effectiveness of the government, which was a measure that captured perceptions of the quality of a country’s public services, among other things [10]. Furthermore, in the district-level analysis (Rwanda and Tanzania), BHBM shows that increased PEPFAR funding per capita is associated with a decrease in all-cause adult-mortality. The results imply that the effects of PEPFAR had accumulated and reached a detectable, statistically significant level.

Our objective was to assess the robustness of Bendavid’s results and determine why there are differences between BDBM’s results and the results of Duber, Coates, Szekeras, Kaji, and Lewis (DCSKL) (2010). To do this, we replicate the BDBM study in three ways: perform a push-button replication, a pure replication, and a measurement and estimation analysis (MEA). The push-button replication attempted to use the original authors’ supplied code and cleaned data set to reproduce the results printed in the journal; the pure replication tried to reproduce the results using only the cleaned data set and description of methods in the journal article. Lastly, as described in the MEA section, an alternative approach was used for the analysis. By replicating the study we provide confirmation of whether the original authors’ work can be reproduced and whether different methods might produce a different interpretation and thus require a re-assessment of policies. By successfully replicating influential studies such as BHBM, we can be even more confident that the policies relying on these studies are based on robust evidence [11].

Our paper is organized as follows. Section 2 describes the methods used for the pure replication and MEA. Section 3 explores the results for both the pure replication and MEA. Following is a discussion in Section 4, and the limitations of the replication study in Section 5 and ending with a conclusion in Section 6. Appendix B Table in S1 File contains all the variables used for our analysis. Appendix C Figure shows the time frame of the studies and key PEPFAR events, and Appendix D Table in S1 File is the summary for comparison of [3] and [4]. Please see [12–16] for more details about the analysis plan and reports at different stages. Please see [17] for the significance of replications in policymaking.

## 2. Methods

### Data and pure replication methods

The BHBM [3] data set is from the Demographic and Health Survey (DHS) data [1]. The de-identified merged person-level data consists of 38 DHS data sets that span 27 African countries, with 9 focus countries and 18 non-focus countries, between 1998 and 2008, as described in BHBM. Using the raw data supplied by the original authors, we independently created a longitudinal data set with repeated observations for the siblings of the respondents in the same way as BHBM. We merged this newly created longitudinal data set with two other data sets provided by the original authors. The two additional data sets contain country-level covariates. The merged data set was used for analysis. See Appendix B for variables that are included in each data set.

Prior to the main analysis, BHBM compares characteristics of PEPFAR and non-PEPFAR countries using two-tailed t-tests and visually examines possible time trends. Specifically, age-adjusted all-cause adult mortality time trend is examined. We implemented the age adjustment using the method described by BHBM; however, BHBM were unclear on the reference population(s) used. Therefore, we used the United Nations Population Division (2015) [18] age-structured population estimates from 2005 for the 27 study countries. For each 5-year age group, we summed the population estimates for the 27 study countries, creating a standard population for each age group. The weight for each age group is the standard population of the age group divided by the sum of the standard populations. The age-adjusted rate is the crude mortality rate for a particular age group multiplied by the appropriate weight and normalized per 1,000. Summing the individual age-adjusted rates gave the age-adjusted mortality per 1,000 for adults aged 15 to 59 years. We calculated age-adjusted rates separately for focus and non-focus countries. We calculated the 95 percent CIs separately for the focus and non-focus countries using a method developed in [19], i.e., rates were assumed to be distributed as a weighted sum of independent Poisson random variables.

The primary analysis of BHBM uses logistic regression with a difference-in-difference indicator to evaluate the effects of PEPFAR implementation. Specifically, the original authors compare the odds of adult (defined as men and women aged 15 to 59 years) all-cause mortality in focus and non-focus countries pre- and post-PEPFAR implementation. They define PEPFAR implementation as post-2003 and compare all-cause mortality at the individual-level using a logistic regression model [3]. Mortality is a binary variable indicating whether or not an adult who was alive for any part of a year died during the year of observation. By examining all-cause mortality at the person level, BHBM were able to adjust the models for individual- and country-level covariates. BHBM used three regression models: unadjusted, country-level adjusted, and individual- and country-level adjusted. All models include year and country fixed effects. Country-level covariates are HIV prevalence, per capita development assistance, GDP, and index of government effectiveness. Individual-level effects include sibling age in years, recall period between the year of the survey and the year of observation, and the respondent’s education and place of residence.

Prior to the main analysis, all-cause age-adjusted mortality trends were visually examined.

After the main analysis, BHBM uses their model to predict deaths averted due to PEPFAR funding using a three-stage process. We used the same three-stage process of BHBM to predict deaths averted due to PEPFAR implementation. We first used our results from the logistic regression adjusted for country- and individual-level covariates to predict two quantities for each person-year observation: the predicted probability of death of a person if PEPFAR had been in place and the predicted probability of death of an individual if PEPFAR had not been in place. We obtained 10 predicted quantities for each observation, two predictions per year for 2004 to 2008. We then calculated (1) the predicted probabilities by focus country and year, (2) the effects of PEPFAR on the decrease in the mortality rate in each year and each focus country, i.e. the difference between the probability of death with and without PEPFAR, (3) the number of deaths averted by focus country by extrapolating the results to the entire population of 15–59 year olds using the United Nations Population Division (2015) [18] age-structured population estimates.

We conducted the replication analysis using the same methods as BHBM using SAS/STAT software version 9.4 (SAS Institute Inc., Cary, NC, USA) and Stata version 14.1. We used the SAS SURVEYLOGISTIC procedure for this analysis. The SURVEYLOGISTIC procedure allows for clustering by countries (i.e., robust/clustered/sandwich standard errors), thereby relaxing the assumption of independent and identically distributed errors within a country. Furthermore, clustered standard errors are a more conservative approach, thus helping to demonstrate the robustness of the model. This methodology permits the computation of unadjusted and adjusted ORs.

We obtained the data provided by BHBM in Stata and converted it to SAS using Stata version 14.1. If there was a discrepancy in the replication results using SAS software, we compared the results to the push-button replication results. Overall, we identify some discrepancies; however, these discrepancies do not have an impact on the main findings. We highlight any differences in the respective tables or figures.

### MEA methods

DCSKL examines the effects of PEPFAR in Africa using 14 health indicators from publicly available data. Health indicators for 46 African countries were collected for 2000 and 2006, using the World Health Organization database. As in BHBM [3], DCSKL examines whether PEPFAR had a greater effect on decreasing all-cause mortality in focus countries, compared with non-focus countries. DCSKL examines the median fractional change in all-cause mortality from 2000 to 2006. They do not find a statistically significant effect when comparing the median fractional change from 2000 to 2006 in all-cause mortality between focus and non-focus countries. These results contradict the findings from BHBM. However, DCSKL and BHBM use different statistical methods, time frames, and countries for their analyses.

We could not directly use BHBM’s methods on the data from DCSKL (See http://www.who.int/whosis/data/Search.jsp) since the structures of the two data sets are different. The BHBM method requires individual-level longitudinal data; whereas, the data used by DCSKL is population-level longitudinal data. Therefore, we utilize BHBM’s methods and data with the DCSKL study period to see if the results are consistent.

We used a subset of the BHBM data set, examining only observations between 2000 and 2006 (inclusive). As in the pure replication, we compared characteristics of the focus and non-focus countries with each other, using a two-tailed t-test. Next, we examined the difference in the odds of all-cause mortality between focus and non-focus countries, using logistic regression with the difference-in-difference indicator. We used the same three regression models as in the pure replication–unadjusted, adjusted for country covariates, and adjusted for country and individual-level covariates. Lastly, we performed a sensitivity analysis similar to the sensitivity analysis carried out in the pure replication. We performed a logistic regression on the unadjusted and adjusted (country- and individual-level covariates) model, leaving out one country at a time, including only countries that had all data for the study period, and using a linear time trend, as opposed to a dichotomous indicator, for PEPFAR implementation.

## 3. Results

### Pure replication results

Our pure replication began by reproducing Table 1 of the original paper [3]. In Table 1, we show, as the original paper did, a summary of the survey fieldwork dates, number of respondents, number of observations after the creation of the longitudinal data set and number of deaths by country. We also stratified the countries by focus and non-focus countries according to the stratification used in the original paper. We show that there were some minor discrepancies in the survey fieldwork dates, but we were able to replicate the number of observations and number of deaths with no discrepancies.

**Table 1:** Replication results of study countries, participants and group designation.

			No. of		
			unique	Observations,	No. of
Country		Survey fieldwork dates	adults	no.	deaths
Focus countries					
	Ethiopia	2–6/2000, 4–8/2005	96,980	391,835	2,596
	Kenya	4–9/2003, 11/2008–3/2009	73,580	491,521	2,971
	Mozambique	8/2003–1/2004	41,103	189,752	1,367
	Namibia	9–12/2000, 11/2006–3/2007	64,382	340,338	3,303
	Nigeria	6–11/2008	122,815	1,020,435	4,590
	Rwanda	5–11/2000, 2–8/2005	74,818	316,179	2,943
	Tanzania	10/2004–2/2005, 12/2009–5/2010	83,992	615,367	2,993
	Uganda	9/2000–3/2001, 5–10/2006	62,132	301,234	2,856
	Zambia	11/2001–6/2002, 4–10/2007	60,014	328,837	4,228
Non-focus countries					
	Benin	8–11/2006	64,463	449,155	1,703
	Burkina Faso	1–3/1999, 6–12/2003	55,416	206,068	1,123
	Cameroon	2–9/2004	41,422	222,637	1,550
	Chad	7–12/2004	20,891	111,943	736
	Congo	7–11/2005	28,305	175,576	1,323
	Congo Dem Rep	1–9/2007	38,637	295,800	1,887
	Gabon	7/2000–2/2001	22,083	43,671	210
	Guinea	4–8/1999, 2–6/2005	44,848	177,877	977
	Lesotho	9/2004–2/2005, 10/2009–1/2010	47,185	334,908	4,428
	Liberia	12/2006–4/2007	23,052	178,489	842
	Madagascar	11/2003–6/2004, 11/2008–7/2009	107,869	844,146	3,509
	Malawi	7–11/2000, 1/2004, 9/2004–2/2005	84,041	305,436	3,945
	Mali	1–6/2001, 3–12/2006	92,775	470,612	2,161
	Niger	1–6/2006	34,858	243,442	942
	Senegal	1–6/2005	55,881	347,114	1,096
	Sierra Leone	4–8/2008	19,675	165,810	891
	Swaziland	6/2006–3/2007	18,458	128,135	1,739
	Zimbabwe	8–12/1999, 8/2005–2/2006, 4/2006	58,937	247,359	3,394

Highlighted cells are those discrepant to the original findings.

Similarly, we replicated Table 2, country-level summary statistics (i.e., population, HIV prevalence, HIV aid in US dollars, etc.) stratified by focus/non-focus country and year. We identify some discrepancies in the CIs that appear to be a result of rounding. However, the point estimate for *HIV aid per country*, *millions of $* for the focus countries for 1998 differ and could not be explained by rounding. This discrepancy led to a difference in p-values comparing focus and non-focus countries for that particular year. We are unsure about the cause of the discrepancy. Furthermore, the push-button replication (PBR), which used the original authors’ supplied code and data with no modification to either, matched our results for *HIV aid per country*, *millions of $* for 1998.

**Table 2:** Replication results of comparison of focus countries and non-focus countries with each other.

		Mean (95% CI)		
Parameter		Focus countries	Non-focus countries	p-value^a^
Population, millions	1998	33.6 (5.1 to 62.1)	9.8 (4.5 to 15)	0.02
	2008	43.4 (7.8 to 79)	12.8 (5.9 to 19.8)	0.01
HIV prevalence among adults 15–49 y old, %	1998	8.1 (5 to 11.3)	6.5 (2 to 11)	0.62
	2008	7.5 (3.9 to 11.1)	5.8 (1.9 to 9.8)	0.57
GDP per capita, constant $	1998	471.3 (–1.4 to 944.1)	641.8 (98.7 to 1,184.8)	0.67
	2008	629.1 (15.1 to 1243.1)	654.5 (180.3 to 1,128.8)	0.95
HIV aid per country, millions of $	1998	7.3 (1 to 13.6)	2 (–0.2 to 4.3)	0.04
	2008	240.5 (168.7 to 312.3)	24.6 (10.2 to 39.1)	<0.001
HIV aid per adult with HIV, $	1998	3.8 (1.8 to 5.7)	6.3 (0.2 to 12.3)	0.55
	2008	171 (75.8 to 266.3)	76.9 (54.9 to 98.9)	0.01
Urban residence, %	1998	24 (15.8 to 32.3)	33.7 (25.4 to 42)	0.13
	2008	28.1 (19 to 37.2)	38 (29.1 to 46.9)	0.15

Highlighted cells are those discrepant to the original findings.

^a^ p-values represent statistical significance of 2-sided t-test comparing focus and non-focus countries.

**Fig 1:**
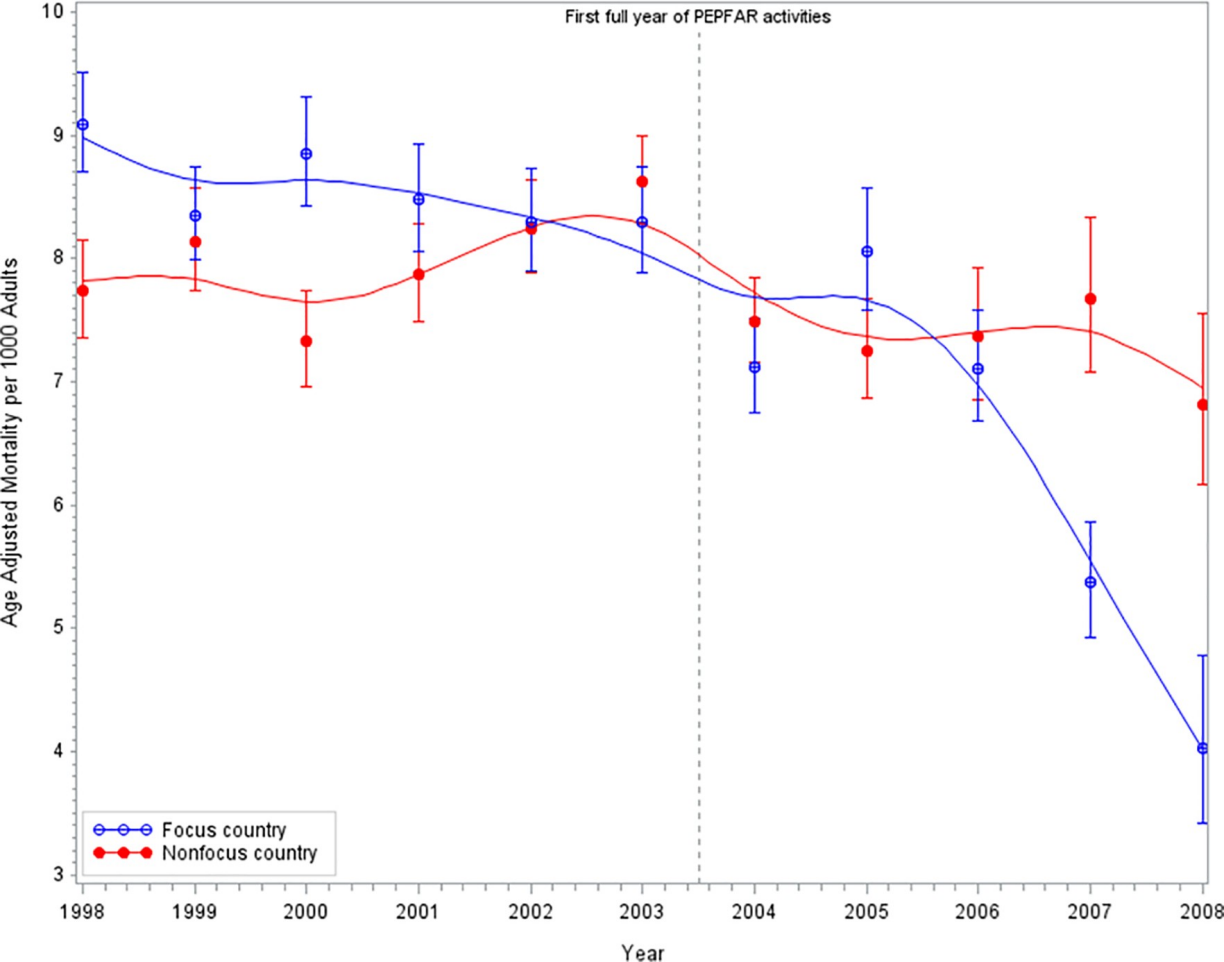
Replication results of age-adjusted mortality trends in the focus and non-focus countries, 1998–2008. Each point represents the probability that an adult aged 15 to 59 years died during the indicated year per 1,000 in either a focus or a non-focus country [3]. The error bars represent 95 percent CIs. Point estimates are age adjusted and age-adjusted CIs are calculated using the method in Fay and Feuer (1997). The trend line is fit by using a smoothing spline.

Next, we reproduced two of the three figures in the original manuscript. We were unable to reproduce Fig 3 of [3], “Adult mortality trends in Tanzania separated by PEPFAR activity, 1998–2008,” as we were unable to obtain the appropriate data. Fig 1 from [3] is a trend in development assistance for HIV to focus and non-focus countries from 1998 to 2008, while Fig 2 from [3] is an age-adjusted adult mortality trend in the focus and non-focus countries for the same years. The original authors considered 2004 to be the first full year of PEPFAR implementation; we indicate the time of full implementation by a vertical dashed line in both figures. Because of copyright concerns, we present only the discordant reproduced figures. Our replication results for Fig 1 of [3] appear to match the original study and is, therefore, not presented again here. Our replication results for Fig 2 from [3] display the same general trend as the original authors; however, the results are not an exact match. We present our corresponding Figure here as Fig 1 below. We hypothesize that the discrepancies in our Fig 1 with Fig 2 of [3] are the result of a different age-adjustment being used.

Our replication results indicate that the age-adjusted adult mortality in 2003 was 8.3 per 1,000 adults (95% CI, 7.9–8.7) in focus countries and 8.6 per 1,000 adults (95% CI, 8.3–9.0) in non-focus countries as shown in Fig 1. This is very similar compared to the original results, which were 8.3 per 1,000 adults in focus countries (95% CI, 8.0–8.6) and 8.5 per 1,000 adults in non-focus countries (95% CI 8.3–8.7). Furthermore, in 2008, our results indicate that age-adjusted adult mortality per 1,000 adults was 4.0 (95% CI, 3.4–4.8) in focus countries and 6.8 (95% CI, 6.2–7.6) in non-focus countries. Whereas the original results for 2008 indicated mortality declined to 4.1 per 1,000 adults in focus countries (95% CI, 3.6–4.6) and 6.9 per 1,000 in non-focus countries (95% CI, 6.3–7.5).

The original results and our results (Fig 1) both show that the age-adjusted mortality in focus countries had been decreasing more rapidly than in non-focus countries after the implementation of PEPFAR. Prior to PEPFAR implementation, focus countries and non-focus countries had similar age-adjusted mortality rates for most years.

We examined the robustness of our results by using different age-adjustments. We used 2000 and 2010 as standard populations and determined that the year used for the standard population did not affect our results. The cause of the discrepancy between our results and the published results is unclear. There is no mention of the year or countries that were used for the standard population in the original paper or the code provided by the original authors. Additionally, we used a different revision of World Population Prospects.

Table 3 of [3] represents the main findings of the original study. We replicated that table (as Table 3 below) with minor discrepancies that could be explained by rounding except in two cases. The 95% CIs for the adjusted models using individual-level covariates for adult death and non-PEPFAR assistance do not match. Our results for the odds of adult death is 0.84, 95% CI (0.72–0.97) compared to 0.84, 95% CI (0.72–0.99) in the original study. Similarly, our results for the odds of non-PEPFAR assistance is 1.00, 95% CI (0.99–1.01) compared to the original odds 0.99, 95% CI (0.96–1.02). These discrepancies do not change the main findings; i.e., PEPFAR funding is associated with a decrease in all-cause adult mortality. Additionally, government effectiveness (odds 0.58, 95% CI, 0.38–0.89) and respondents’ education level (odds 0.99, 95% CI, 0.98–1.00) are associated with a decrease in all-cause adult mortality.

**Table 3:** Replication results for regression models estimating the odds ratios ratio of death in study adults in focus countries versus non-focus countries.

	Unadjusted OR (95% CI)^a^	p-value	Adjusted OR with country covariates (95% CI)	p-value	Adjusted OR with country and personal covariates (95% CI)	p-value
Adult death^b^	0.80 (0.68–0.95)	0.01	0.82 (0.72–0.95)	0.01	0.84 (0.72–0.97)	0.02
HIV prevalence (per additional 1%)			1.07 (1.00–1.15)	0.04	1.07 (1.01–1.14)	0.03
Non-PEPFAR assistance^c^			1.00 (0.98–1.01)	0.87	1.00 (0.99–1.01)	0.89
GDP per capita (per additional $1)			1.00 (1.00–1.00)	0.82	1.00 (1.00–1.00)	0.63
Government effectiveness (per 1 point increase)^d^			0.62 (0.40–0.95)	0.03	0.58 (0.38–0.89)	0.01
Sibling age (per year)					1.05 (1.04–1.05)	<0.001
Residence in urban area^e^					0.94 (0.89–1.00)	0.05
Education (per additional year)					0.99 (0.98–1.00)	0.01
Recall (interval between survey and observation, per year)					0.97 (0.95–0.99)	0.01

Highlighted cells are those discrepant to the original findings.

^a^ All results are the estimated odds ratios (OR) and 95% confidence intervals (CI). All CIs are estimated using robust standard errors. The unadjusted model includes country and year covariates.

^b^ These ORs represent the odds of all-cause adult mortality among individuals living in focus countries compared to individuals living in non-focus countries during the implementation of PEPFAR. All-cause adult mortality was a dichotomous variable measured for each individual in the study.

^c^ All health-related assistance less US-provided HIV assistance per capita.

^d^ Government effectiveness is standardized, i.e., each point increase represents an increase of 1 standard deviation. Higher numbers indicated increased government effectiveness.

^e^ These variables are gathered from the interviewee and do not necessarily represent characteristics of the sibling.

Next, we examined the number of deaths averted by PEPFAR. We find that our results are reasonably consistent with the original results, except for Mozambique and Rwanda (eTable 3 in S1 File). Point estimates for these two countries differ by a large margin. These different point estimates then affected the calculation of deaths averted.

We are not surprised that our results do not exactly match the original results. When reproducing the main results (i.e., Table 3), it was clear that our logistic regression had slightly different coefficients, which would affect the predicted mortalities. Additionally, the original authors’ process was somewhat ambiguous. We were unsure how they predicted their mortalities from the logistic regression. Based on their description, it appears that they predicted only two quantities (mortality with PEPFAR and mortality without PEPFAR) and then limited the predictions to the years 2004 to 2008. However, this would not be possible, since Mozambique did not have any surveys administered during this period. It is also unclear what standard population (as previously mentioned) the original authors use. These differences aside, we feel that our replication results support the findings of the original authors for the number of deaths averted.

To examine the robustness of the results, the original authors performed several sensitivity analyses as described above (MEA methods). We completed the same sensitivity analyses as the original authors. We examined whether any one country unduly affected the main findings by conducting a leave-one-out analysis. We performed the analysis leaving one country out at a time to determine if one country was leveraging the results. Our results are in agreement with the original results; the magnitude and direction of the ORs appear consistent when performing the leave-one-out analysis. There seem to be some rounding errors, which we do not highlight. There are four cases where rounding could not explain the discrepancy, which we do highlight. These differences do not change the significance of the results. We display our results in eTable 4 in S1 File, along with the original results and notes.

To examine further the impact specific countries might have on the results, three subsets of countries were created as per the original sensitivity analysis. Using these subsets of countries, an unadjusted and two adjusted logistic regressions were performed, as did the original authors. The first subset of countries comprises those with data before and after PEPFAR implementation: Benin, Congo, Democratic Republic of Congo, Ethiopia, Guinea, Kenya, Lesotho, Liberia, Madagascar, Malawi, Mali, Namibia, Niger, Nigeria, Rwanda, Senegal, Sierra Leone, Swaziland, Tanzania, Uganda, Zambia and Zimbabwe. The second subset uses only the most recent survey for each country. The third subset uses only countries with data from 1998 through at least 2007, which consists of four focus countries (Kenya, Nigeria, Tanzania, and Zambia) and three non-focus countries (Lesotho, Madagascar, and Sierra Leone).

There are multiple differences between our results and the original results. We only highlight differences if the OR switched from significant to non-significant and vice versa. Our results from this sensitivity analysis further support the robustness of the main findings of BHBM. We show our sensitivity analysis results and the original sensitivity analysis results and notes in Table 5 in S1 File.

The last sensitivity analysis performed by the original authors repeated the main analysis, using a linear time trend instead of a binary indicator for the main variable of interest. This analysis might be underpowered, but it would show if the general trend held–i.e., if PEPFAR was associated with a decrease in all-cause adult mortality. As with the previous sensitivity analysis, our results show multiple discrepancies when compared with the original results. Again, we only highlight results that changed from significant to non-significant and vice versa. Under the fully adjusted regression model, our results indicate that the main variable of interest is now borderline significant; however, there is some loss of power when using a linear time trend. The direction of the ORs is consistent, and we feel that our results still show the robustness of the findings. We display our results in eTable 6 in S1 File, along with the original results and notes from the original authors.

It is unclear why our point estimates and the CIs differ in the last two sensitivity analyses. We created our longitudinal data set from the cleaned data set provided by the authors. Additionally, the results of the PBR match the results that we report in this section. Furthermore, the PBR was not able to produce the subnational analysis using district-level data for Tanzania and Rwanda since the data were not made available.

### MEA results

For the MEA, group comparisons show that the characteristics of the focus and non-focus countries are similar in most respects, except population and HIV aid per country. The mean population of the focus countries is statistically larger than the mean population of the non-focus countries, regardless of the year examined. Additionally, in 2000 and 2006, focus countries received significantly more aid (in millions of dollars) than non-focus countries. In 2000, the mean HIV aid in focus countries was US$16.6 million (95% CI, 8.3–24.9), rising to US $125.8 million (95% CI, 97–154.6) in 2006. Non-focus countries had an increase in aid from 2000 to 2006, but the increase was quite small compared with focus countries. In non-focus countries, mean aid changed from US $4.8 million (95% CI, 1.2–8.3) to US $17.6 million (95% CI, 8–27.1). However, when examining aid per adult with HIV, the difference between focus and non-focus countries is not significant, regardless of the year. Mean aid per adult living with HIV in 2000 was US $10 in focus countries (6.2, 13.7) versus US $26.9 in non-focus countries (–2.3, 56.1). Furthermore, group comparisons show that mean HIV prevalence among adults 15 to 49 years old in 2000 was not statistically different between focus and non-focus countries (p-value 0.67). The mean prevalence of HIV in focus countries was 8.1 percent (95% CI, 4.8–11.5) as opposed to 6.7 percent (95% CI, 2.1–11.3) for non-focus countries. See Table 4 for the remaining results.

**Table 4:** Comparison of focus and non-focus countries with each other for 2000 and 2006.

		Mean (95% CI)		
Parameter		Focus countries	Non-focus countries	p-value^a^
Population, millions	2000	34.8 (6.2 to 63.3)	10.4 (4.9 to 16)	0.02
	2006	40.9 (7.6 to 74.2)	12.2 (5.5 to 18.8)	0.01
HIV prevalence among adults	2000	8.1 (4.8 to 11.5)	6.7 (2.1 to 11.3)	0.67
15–49 years old, %	2006	7.7 (3.9 to 11.5)	6.1 (2.1 to 10.1)	0.59
GDP per capita, constant $	2000	480.8 (–2.5 to 964)	609.6 (143.7 to 1,075.5)	0.71
	2006	586.9 (3 to 1170.8)	634.5 (177.7 to 1,091.2)	0.89
HIV aid per country, millions of $	2000	16.6 (8.3 to 24.9)	4.8 (1.2 to 8.3)	0.002
	2006	125.8 (97 to 154.6)	17.6 (8 to 27.1)	<0.0001
HIV aid per adult with HIV, $	2000	10 (6.2 to 13.7)	26.9 (–2.3 to 56.1)	0.40
	2006	104.8 (38.9 to 170.7)	66.5 (42 to 91)	0.15
Urban residence, %	2000	24.8 (16.6 to 33)	34.5 (26 to 43)	0.13
	2006	27.3 (18.5 to 36.1)	37.1 (28.3 to 45.9)	0.15

^a^ p-values represent statistical significance of 2-sided t-test comparing focus and non-focus countries.

Our unadjusted regression analysis indicates that the odds of all-cause mortality after PEPFAR implementation (2003) for individuals living in focus countries was 0.86 (95% CI, 0.74–1.00; p-value 0.04) compared with people living in non-focus countries. This statistically significant reduction in the odds of adult mortality holds in the adjusted model with country covariates but does not for the adjusted model with country- and individual-level covariates. When examining the adjusted model with country- and individual-level covariates, the OR of death is 0.87 (95% CI, 0.75–1.01; p-value 0.07). Additionally, regardless of the model, an increase in HIV prevalence is not associated with a change in the OR of all-cause adult mortality. Of the remaining covariates in the fully adjusted model, only sibling age and education (per additional year) are associated with all-cause adult mortality. For each year increase in sibling age, the OR of death is 1.05 (95% CI, 1.04–1.05; p-value <0.01). Each additional year of education is protective, with an associated OR of death of 0.99 (95% CI, 0.98–1.00; p-value <0.01). Table 5 contains the full results from all regression models.

**Table 5:** Regression models estimating the odds of death in study adults in focus versus non-focus countries for 2000–2006.

	Unadjusted OR (95% CI)^a^	p-value	Adjusted OR with country covariates (95% CI)	p-value	Adjusted OR with country and personal covariates (95% CI)	p-value
Adult death^b^	0.86 (0.74–1.00)	0.04	0.86 (0.76–0.97)	0.02	0.87 (0.75–1.01)	0.07
HIV prevalence (per additional 1%)			1.04 (0.98–1.10)	0.16	1.05 (0.99–1.10)	0.08
Non-PEPFAR assistance^c^			0.99 (0.97–1.00)	0.14	0.99 (0.97–1.00)	0.09
GDP per capita (per additional $1)			1.00 (1.00–1.00)	0.28	1.00 (1.00–1.00)	0.50
Government effectiveness (per 1 point increase)^d^			0.81 (0.60–1.08)	0.16	0.76 (0.53–1.08)	0.12
Sibling age (per year)					1.05 (1.04–1.05)	0.00
Residence in urban area^e^					0.95 (0.88–1.02)	0.18
Education (per additional year)^e^					0.99 (0.98–1.00)	0.00
Recall					0.97 (0.94–1.00)	0.05

^a^ All results are the estimated odds ratios (OR) and 95% confidence intervals (CI). All CIs are estimated using robust standard errors. The unadjusted model includes country and year covariates.

^b^ These ORs represent the odds of all-cause adult mortality among individuals living in focus countries compared to individuals living in non-focus countries during the implementation of PEPFAR. All-cause adult mortality was a dichotomous variable measured for each individual in the study.

^c^ All health-related assistance less US-provided HIV assistance per capita.

^d^ Government effectiveness is standardized, i.e., each point increase represents an increase of 1 standard deviation. Higher numbers indicated increased government effectiveness.

^e^ These variables are gathered from the interviewee and do not necessarily represent characteristics of the sibling.

We further examine the sensitivity of these results by leaving any one country out from the regression models. The results appear robust. For the unadjusted model, the direction and magnitude of the point estimates are consistent, and the majority of the point estimates are statistically significant. The non-significant point estimates are non-significant by a small margin. Results for the fully adjusted regression model are similar, except that most point estimates remained statistically non-significant, as in the original results. See Table 6 of [16].

When examining only the countries where data is available for all years from 2000 to 2006, the original findings hold. The unadjusted and adjusted with country covariates regression models indicate that PEPFAR is statistically protective against all-cause adult mortality, whereas there is not a significant association between all-cause adult mortality and PEPFAR when examining the regression model with country- and individual-level covariates. HIV prevalence remains a statistically non-significant indicator of all-cause mortality with an OR of 1.06 (95% CI, 0.99–1.14) when examining countries with complete data for 2000 to 2006. As with the original findings, each additional year of education is protective, with an OR of 0.99 (95% CI, 0.98–1.00), and sibling’s age (per year) increases the odds of all-cause mortality, with an OR of 1.05 (95% CI, 1.04–1.05). However, in the original results for 2000 to 2006, government effectiveness was not significantly associated with all-cause mortality (OR 0.76; 95% CI, 0.53–1.08). When limiting the data set to countries with data for all years from 2000 to 2006, government effectiveness is significantly associated with a decreased odds of all-cause mortality (OR 0.59; 95% CI, 0.46–0.76). See Table 7 of [16]. The focus countries with complete data are Kenya, Namibia, Nigeria, Tanzania, and Zambia; non-focus countries are Democratic Republic of Congo, Lesotho, Liberia, Madagascar, Sierra Leone, and Swaziland.

The last sensitivity analysis performed was a linear time trend. All three regression models (one unadjusted model and two adjusted models) indicate that PEPFAR is associated with a reduction in the odds of all-cause mortality using a linear time trend; however, these point estimates are all non-significant, though they all show consistent direction and magnitude. As with the original 2000 to 2006 results, HIV prevalence is not associated with all-cause mortality (OR 1.05; 95% CI, 0.99–1.11). Additionally, sibling age (per year) remains associated with all-cause mortality (OR 1.05; 95% CI, 1.04–1.05) and education (per additional year) offers protection against all-cause mortality (OR 0.99, 95% CI, 0.98–1.00). See Table 8 of [16].

## 4. Discussion

In this paper, we perform a replication and comparative analysis on the paper “HIV Development Assistance and Adult Mortality in Africa” to verify the original results and asses their robustness. This then allows us to draw conclusions about why there are differences between BHBM [3] and Duber et al.[4]. Using the paper and the electronic appendix as a guide, we were able to replicate the results, excluding the subnational analysis of district-level data for Tanzania and Rwanda.

In the comparative analysis, we aimed to examine how the choice of time and countries would affect the results of DCSKL’s paper [4], in which the authors find no statistical evidence that PEPFAR influenced the adult mortality rate when comparing focus and non-focus countries (p-value 0.348). These results are not in agreement with an earlier paper by Bendavid and Bhattacharya [6] and the current paper by BHBM [3]. Using the method from [3] and the study period from [4], we find that PEPFAR had a significant impact on all-cause adult mortality when examining the effects of PEPFAR using an unadjusted and adjusted logistic regression with country-level covariates.

The fully adjusted model (with country- and individual-level covariates) is just outside the bounds of significance when examining the association between PEPFAR and all-cause adult mortality. The results for the fully adjusted model are not surprising, since the sample size is smaller, which would widen the CIs. However, the point estimate for the 2000 to 2006 data did fall within the CI of the original analysis and is close to the original point estimate in [3]. A possible explanation for the non-significant results of DCSKL [4] was that PEPFAR activity requires a sufficient amount of time for the effects to accumulate in the focus countries. If this assertion were true, then we would expect more varied point estimates in the fully adjusted models using BHBM’s method for the restricted data (2000 to 2006) and the original analysis (1998 to 2008). However, we did not see these from our study.

The two studies used different focus countries for analysis. DCSKL [4] included three additional countries (Botswana, South Africa, and Cote d’Ivoire) that BHBM [3] did not include. BHBM stated that these countries were not included due to unsuitable data sources. Furthermore, Botswana and South Africa had particularly high HIV-prevalence that could affect the results of the pure replication and MEA. DCSKL stated that it appeared that South Africa showed worsening health indicators during the study period. It is possible that not including South Africa as a focus country biased the results of the pure replication and MEA.

Lastly, we were unable to obtain district-level data for Tanzania and Rwanda to replicate the effects of the amount of PEPFAR related activity on all-cause adult mortality. While having access to district-level data for Tanzania and Rwanda would have allowed us to assess this part of the original study findings, we were unable to make an assessment about the intensity of PEPFAR and its impact on all-cause mortality. However, the original authors were able to obtain the data and show that higher levels of PEPFAR related activity is associated with a significant decrease in all-cause adult mortality. In BHBM for both the main analysis and sub-analysis, the primary intervention was dichotomously defined. And by using a more nuanced or continuous surrogate for PEPFAR implementation, e.g., percent of antiretroviral coverage, amount of HIV funding, etc., might provide additional support for PEPFAR’s overall effect; however, it was beyond the scope of this paper to create an index to define PEPFAR intensity.

Due to a lack of individual-level indicators in the Duber data, the different statistical methods used by BHBM and Duber, along with the different focus countries between the two studies, it was difficult to untangle the major cause for the discrepant results of [3] and [4]. We did find, however, that in the focus countries used by BHBM, PEPFAR was associated with a decrease in all-cause adult mortality in a short time frame.

## 5. Limitations

We excluded Botswana and South Africa from the analysis, as did by BHBM [3]. Not including Botswana and South Africa in the analysis could affect the significance of the main findings, as both countries had a high prevalence of HIV. If PEPFAR was reducing all-cause mortality by reducing HIV-related mortality, then including Botswana and South Africa might strengthen BHBM’s main findings. While we had hoped to update the analysis with more recent data, this was not done due to limitations in resources and recommendations from an external proposal reviewer. However, we show that the results in BHBM [3] are robust and that PEPFAR is associated with the reduction in all-cause mortality between focus and non-focus countries as defined by BHBM [3].

## 6. Conclusions

DCSKL [4] and BHBM [3] are not in agreement about the effectiveness of PEPFAR in reducing all-cause adult mortality. Our replication study supports the findings of BHBM. Our additional analyses are unable to answer whether the methods used by [3] and [4] were the cause of the discordant results. Both papers mention South Africa (not included in BHBM’ data set) as a possible linchpin in their results. The study period did not have a large impact on the results when using the data set of [3].

We cautiously agree with the BHBM [3] findings that PEPFAR is associated with a reduction in all-cause adult mortality in focus countries compared to non-focus countries for 2004 to 2008. We are unable to state whether the decrease in all-cause mortality was because of a reduction in HIV mortality or some other mechanism. All these are interesting topics to be explored in the future.

## Supporting information

S1 File Contains Appendices A-D, supporting tables and figures.
(DOCX)
