# Supplementary material for: The President’s Emergency Plan for AIDS Relief and adult mortality: A replication study of HIV development assistance effects in Sub-Saharan African countries
Source: PLoS One. 2020 Oct 26;15(10):e0233948. doi: 10.1371/journal.pone.0233948 (PMC7588097; doi:10.1371/journal.pone.0233948)
Supplement: S1 File — Contains Appendices A-D, supporting tables and figures. (DOCX) [file pone.0233948.s001.docx]

# Appendix A: Tables

eTable 3: Replication results for estimation of the number of deaths averted for the period 2004 – 2008

|  | Adult mortality with PEPFAR* | Adult mortality without PEPFAR** | Adult population (millions)^+^ | Deaths averted (thousands)^++^ |
| --- | --- | --- | --- | --- |
| Ethiopia | 5.4 | 6.5 | 38.8 | 201.7 |
| Kenya | 5.2 | 6.2 | 19.3 | 96.2 |
| Mozambique | 5.9 | 7.0 | 10.7 | 60.3 |
| Namibia | 8.2 | 9.8 | 1.1 | 9.0 |
| Nigeria | 3.9 | 4.7 | 74.2 | 280.2 |
| Rwanda | 7.6 | 9.1 | 5.0 | 36.2 |
| Tanzania | 4.2 | 5.0 | 20.3 | 82.2 |
| Uganda | 7.9 | 9.4 | 13.6 | 102.0 |
| Zambia | 10.8 | 12.9 | 6.1 | 62.3 |
| Total |  |  |  | 930.1 |

Notes:

Highlighted cells are those discrepant to the original findings.

* These are the average model estimated annual all-cause adult mortality per 1,000 adults from 2004-2008 assuming PEPFAR was implemented in the focus countries (“actual” scenario).

** These are the average model estimated annual all-cause adult mortality per 1,000 adults from 2004-2008 assuming PEPFAR had not been implemented in the focus countries (“counterfactual” scenario).

^+^ Mean adult population between 2004 to 2008.

^++^ Deaths adverted is calculated as the difference between the “actual” and “counterfactual” scenario multiplied by the size of the adult population. This calculation is done for each year between 2004-2008 and is then summed.

eTable 4: Replication results for leave-one-out analysis

| Country left out | Unadjusted OR (95% CI) | Adjusted OR  (95% CI)* |
| --- | --- | --- |
| Benin | 0.80 (0.67–0.94) | 0.83 (0.71–0.95) |
| Burkina Faso | 0.80 (0.68–0.95) | 0.84 (0.72–0.97) |
| Cameroon | 0.80 (0.68–0.95) | 0.84 (0.72–0.97) |
| Chad | 0.80(0.68–0.95) | 0.84 (0.73–0.97) |
| Congo | 0.79 (0.67–0.94) | 0.82 (0.71–0.95) |
| Congo Dem Rep | 0.80 (0.67–0.95) | 0.84 (0.72–0.98) |
| Ethiopia | 0.82 (0.69–0.97) | 0.86 (0.74–1.00) |
| Gabon | 0.80 (0.68–0.95) | 0.85 (0.74–0.99) |
| Guinea | 0.80 (0.67–0.94) | 0.83 (0.72–0.97) |
| Kenya | 0.80 (0.67–0.96) | 0.82 (0.70–0.96) |
| Lesotho | 0.86 (0.74–0.99) | 0.89 (0.78–1.02) |
| Liberia | 0.80 (0.67–0.95) | 0.84 (0.72–0.97) |
| Madagascar | 0.79 (0.66–0.94) | 0.84 (0.71–0.99) |
| Malawi | 0.79 (0.67–0.94) | 0.84 (0.73–0.98) |
| Mali | 0.80 (0.67–0.95) | 0.85 (0.73–0.99) |
| Mozambique | 0.80 (0.68–0.95) | 0.84 (0.72–0.97) |
| Namibia | 0.78 (0.65–0.93) | 0.82 (0.70–0.96) |
| Niger | 0.79 (0.67–0.94) | 0.83 (0.72–0.96) |
| Nigeria | 0.76 (0.64–0.90) | 0.80 (0.68–0.93) |
| Rwanda | 0.83 (0.70–0.97) | 0.84 (0.72–0.98) |
| Senegal | 0.80 (0.67–0.94) | 0.83 (0.72–0.96) |
| Sierra Leone | 0.81 (0.68–0.96) | 0.85 (0.73–0.99) |
| Swaziland | 0.81 (0.69–0.96) | 0.83 (0.70–0.97) |
| Tanzania | 0.81 (0.68–0.96) | 0.84 (0.71–0.98) |
| Uganda | 0.82 (0.69–0.97) | 0.86 (0.74–0.99) |
| Zambia | 0.80 (0.67–0.97) | 0.84 (0.71–0.99) |
| Zimbabwe | 0.79 (0.67–0.94) | 0.85 (0.73–0.99) |

Note:

Highlighted cells are those discrepant to the original findings.

eTable 5: Replication results for relative odds of death associated with PEPFAR for subsets of countries and surveys

|  | Unadjusted  (95% CI, p-value)^a^ | Adjusted with country covariates  (95% CI, p-value) | Adjusted with personal and country covariates  (95% CI, p-value) |
| --- | --- | --- | --- |
| Subset (i)^b^ | 0.80 (0.68–0.95, 0.01) | 0.83 (0.73–0.95, 0.01) | 0.85 (0.74–0.99, 0.03) |
| Subset (ii)^b^ | 0.90 (0.76–1.08, 0.27) | 0.94 (0.79–1.13, 0.52) | 0.94 (0.78–1.13, 0.51) |
| Subset (iii)^b^ | 0.75 (0.60–0.93, 0.01) | 0.73 (0.61–0.88, 0.001) | 0.72 (0.59–0.86, 0.0004) |

Notes:

Highlighted cells are those discrepant to the original findings.

^a^ Unadjusted model includes country and year covariates. All 95% CIs are estimated using robust standard errors.

^b^ These ORs represent the odds of all-cause adult mortality among individuals living in focus countries compared to individuals living in non-focus countries during the implementation of PEPFAR. All-cause adult mortality was a dichotomous variable measured for each individual in the study.

(i) Includes countries with survey data before and during the implementation of PEPFAR.

(ii) Uses only the most recent survey data.

(iii) Includes countries with survey data from 1998 through 2007 or beyond.

eTable 6: Replication results for sensitivity analysis using linear time trends

|  | Unadjusted  (95% CI, p-value)^a^ | Adjusted with country covariates  (95% CI, p-value) | Adjusted with country and personal covariates (95% CI, p-value) |
| --- | --- | --- | --- |
| Adult death^b^ | 0.93 (0.86–0.99, 0.04) | 0.94 (0.89–1.00, 0.05) | 0.94 (0.88–1.00, 0.06) |
| HIV prevalence (per additional 1%) |  | 1.08 (1.00–1.16, 0.04) | 1.07 (1.00–1.14, 0.04) |
| Non-PEPFAR assistance^c^ |  | 1.00 (0.98–1.01, 0.79) | 1.00 (0.99–1.01, 0.95) |
| GDP per capita (per additional $1) |  | 1.00 (1.00–1.00, 0.66) | 1.00 (1.00–1.00, 0.54) |
| Government effectiveness (per 1 point increase)^d^ |  | 0.61 (0.39–0.94, 0.03) | 0.57 (0.37–0.88, 0.01) |
| Sibling age (per year) |  |  | 1.05 (1.04–1.05, <0.001) |
| Residence in urban area |  |  | 0.94 (0.89–1.00, 0.05) |
| Education (per additional year) |  |  | 0.99 (0.98–1.00, 0.01) |

Notes:

Highlighted cells are those discrepant to the original findings.

^a^ All results are the estimated odds ratios (OR) and 95% confidence intervals (CI). All CIs are estimated using robust standard errors. The unadjusted model includes country and year covariates. .

^b^ These ORs represent the odds of all-cause adult mortality among individuals living in focus countries compared to individuals living in non-focus countries during the implementation of PEPFAR. All-cause adult mortality was a dichotomous variable measured for each individual in the study.

^c^ All health-related assistance less US-provided HIV assistance per capita.

^d^ Government effectiveness is standardized, i.e., each point increase represents an increase of 1 standard deviation. Higher numbers indicated increased government effectiveness.

Appendix B: Variables contained in each data set

| Country-level covariates | | |  | Individual-level covariates |
| --- | --- | --- | --- | --- |
| **Dataset 1** |  | **Dataset 2** |  | **Dataset 3** |
| Country name |  | Country name |  | Country code and phase |
| Year |  | 2 letter country abbreviation |  | Case identification |
| Government effectiveness |  | Year |  | Year of interview |
| Standard error |  | Indicator for focus country |  | Date of interview (cmc) |
|  |  | Population total |  | Type of place of residence |
|  |  | GDP per capita (constant 2000 US$) |  | Education in single years |
|  |  | Urban population (% of total) |  | Unique index woman identifier |
|  |  | Adult HIV prevalence |  | Numeric survey identifier |
|  |  | Total HIV aid from IHME |  | Maternal mortality index for each sibling |
|  |  | Total HIV aid per person |  | Sex of each sibling |
|  |  | Total HIV aid/ plwha |  | Each sibling's survival status |
|  |  | Total HIV aid from the US |  | cmc date of birth of for each sibling |

Appendix C: Figure: Time Frame of Studies and key PEPFAR events


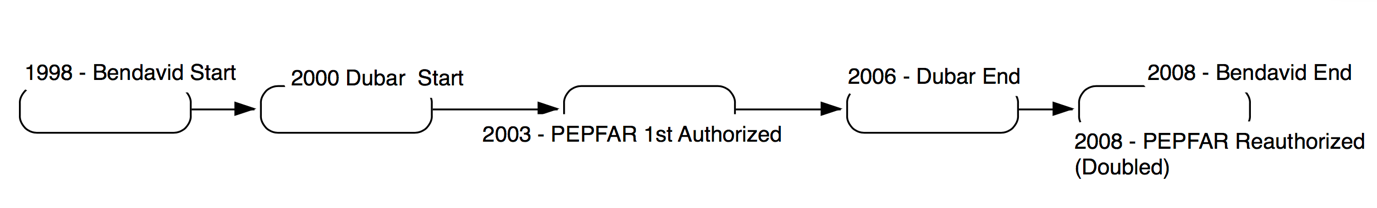


Appendix D: Table. Summary Comparison between Bendavid and Duber Studies

|  | **Bendavid** | **Duber** |
| --- | --- | --- |
| **Scope of analyses (data)** | 1998 through 2008  -        Time series including years between then | 2000 (baseline)  vs 2006 |
| **Data Sources** | DHS –  (Macro Inc)  - strict data collection methodology every 5 years.  -        individual level data set  -        person level covariates  -        country level time-varying covariates  -        data on non-PEPFAR funding indicated | WHOSIS  database (based on country reporting).  -        Aggregated at the country level  -        Only 2 years for which pertinent data  for indicators is available |
| **Primary outcome** | -        All cause Adult mortality (15-59 years) | -        All cause Adult mortality  -        13 other indicators |
| **Analytical methods** | -        Individual level difference in difference analyses  -        Cross-country analysis  -        Within-country analysis  -        Adjustment for person and country level and time Fixed effects  -        Adjustments for time-varying country effects  -        Account of cluster effects  -        Age-adjusted adult mortality  -        Logistic regression methods  -        Odds ratios  (95% Confidence Intervals)  -        Parametric based methods  -        Graphical time-series methods (including lowess curves and error bars)  -        Sensitivity analyses – leave out one methods  -        Intensity (dose-response) analyses in 2 countries with data | -        country level aggregated analyses  -        Fractional changes between 2000 (base) and 2006  (changes normalized to 2000 value)  -        Equal weighting all outcome indicators including adult mortality  -        Median country-level  changes between two time points  (Inter quantile ranges)  -        Non-parametric based methods  -        Statistical  testing based on Wilcoxon signed rank test  -        No adjustments for time or country level covariates  -        No adjustment for multiple comparisons |
| **Sample** | 9 Focus countries vs 18 Non-focus countries  (total sample size for time series  of 1,538,612 adults)  (based on DHS data availability  and inclusion of a Maternal Mortality Module-  South Africa, Botswana  (Macro Inc)  and Cote’ D’voire not included) | All 12 Focus countries vs 34 Non-focus countries |
| **Results with respect to all-cause mortality:**  **Time- Frame 1998-2008:**  **-        Adjusted for personal and country  covariates (Bendavid)**  **-        Adjusted for personal and country  covariates (Replication)** | -        OR(CI) 0.84 (0.72-0.99, 0.03)      -        OR(CI) 0.84 (0.72-0.97,0.02) | -        NA      -        NA |
| **Time Frame 2000-2006:**  **-        Unadjusted**  **-        Adjusted Country covariates**  **-        Adjusted Country and Personal Covariates** | -        OR (CI)  0.84 (0.74 - 0.95, 0.01);  -        OR (CI) 0.88 (0.79 - 0.99,0.04)  ;  -        ;  -        OR (CI)  0.88 (0.78 - 1.01, 0.06) | -        -0.029* (-0.111,0.046,0.348)  -        NA;  -        ;  -        NA |

|  | **Bendavid** | | **Duber** | |
| --- | --- | --- | --- | --- |
| Scope of analyses (data) | Longitudinal (1998 - 2008) | | Change in baseline (2000 vs. 2006) | |
| Data sources | DHS | | WHOSIS | |
|  |  | • Strict data collection methodology |  | • Based on country reporting |
|  |  | • Individual level data |  | • Aggregated at the country level |
|  |  | • Person level covariates |  |  |
| Primary outcome | All cause adult mortality (15-59 years) | | All cause adult mortality | |
|  |  |  | 13 additional indicators | |
| Analytical methods | Logistic regression difference-in-difference analyses | | Fractional changes between 2000 and 2006 | |
|  |  | • Adjusted for person and country level and time fixed effects |  | • Equal weighting amongst the 14 indicators |
|  |  | • Account for cluster effects |  | • No adjustments for time or country level covariates |
|  |  | • Parametric based methods |  | • Non-parametric based methods |
|  |  | • Dose-repsonse sub-analysis |  | • No adjustment for multiple comparisons |
| Sample | 9 focus countries | | 12 focus countries | |
|  |  | • South Africa, Botswana, and Cote’ D’voire not included | 34 non-focus countris | |
|  | 18 non-focus countries | |  |  |
| Results (1998-2008) | Odds ratio 0.84 with 95% onfidence interval (0.72, 0.99) | | NA | |
| Results (2000-2006) | Odds ratio 0.88 with 95% onfidence interval (0.78, 1.01) | | Fraction change -0.029 with 95% confidence interval | |
|  |  |  |  | (-0.111, 0.046) |
